# Music as a tool for personal and social resilience: a three-level conceptual model

**DOI:** 10.3389/fpsyg.2026.1953791

**Published:** 2026-10-01

**Authors:** Ori Leshman

**Affiliations:** Baruch Ivcher School of Psychology, Reichman University, Herzliya, Israel

**Keywords:** autobiographical memory, conceptual analysis, dialogue, emotion regulation, music, music psychology, resilience, social resilience

## Abstract

**Background:**

Music is widely recognized as a resource in times of adversity, yet the mechanisms by which it supports resilience are frequently discussed in fragmented ways. Existing scholarship has highlighted music’s roles in emotion regulation, autobiographical memory, and social connection, but these dimensions are rarely integrated within a single conceptual framework.

**Objectives:**

This article develops an integrative conceptual model explaining how music may support personal and social resilience across structural, intrapsychic, and interpersonal dimensions.

**Conceptual approach:**

Drawing on research in emotion regulation, resilience theory, music psychology, music-evoked autobiographical memory, and prior integrative frameworks in the field, the article advances a three-level conceptual analysis. The model is situated alongside related frameworks in order to clarify its distinctive contribution.

**Models:**

The article develops a three-level conceptual model through which music may contribute to resilience: (1) musical structure, where tension, containment, ambiguity, continuity, and release may function as experiential analogues of adaptive coping; (2) personal meaning, where music becomes linked to autobiographical memory, identity, continuity, and narrative integration, with songs constituting a particularly important case because lyrics may help consolidate and articulate meaning; and (3) dialogical-social sharing, where the exchange of personally meaningful music and the stories associated with it may foster recognition, belonging, prosocial agency, and mutual support.

**Conclusion:**

Music may be understood not merely as an emotional medium, but as a structured experiential space in which individuals and groups can engage capacities central to resilience. The article’s main contribution is a three-level integrative model that explicitly connects temporal musical structure, autobiographical meaning, and dialogical-social sharing within one unified framework of personal and social resilience, while also specifying possible transitions and interactions among these levels.

## Introduction

Resilience is commonly understood as the capacity to cope with adversity, adapt under strain, and maintain or recover psychological functioning in the face of challenge. It is not an inherent attribute of music as an object, but a dynamic human process that music may help support. This distinction is especially important in discussions of music and resilience. The claim of the present article is therefore not that resilience somehow exists in music itself, but that music can serve as a distinctive experiential medium through which resilience-related processes may be modeled, felt, interpreted, and shared.

The central problem addressed here is conceptual fragmentation. Research on music and psychological adaptation often proceeds along parallel lines: some studies emphasize emotion regulation, others autobiographical memory, identity, wellbeing, or social connection. Each of these perspectives contributes something important, yet they are not always integrated within a single explanatory framework. As a result, the field still lacks a sufficiently integrative account of how structural, intrapsychic, and interpersonal aspects of musical experience work together in resilience-related processes.

The main contribution of this article is to develop a three-level conceptual model of music-based resilience. The novelty of the framework lies not simply in identifying structural, autobiographical, and interpersonal dimensions of music, but in theorizing how these domains may become linked within resilience-related processes: from temporally structured affective experience, to personally narrated meaning, to dialogical-social recognition and support. At the first level, music is examined as structured temporal experience, characterized by tension, suspension, movement, ambiguity, containment, and release. At the second level, music is understood as a carrier of personal meaning, linked to autobiographical memory, identity, continuity, and narrative integration. At the third level, music becomes a medium of dialogical-social encounter, through which personally meaningful music and the stories associated with it can be shared in ways that foster recognition, belonging, and mutual support.

This article is written as a conceptual analysis. It does not report new empirical findings; rather, it advances an integrative theoretical model intended to clarify mechanisms, delimit claims, and guide future empirical work on music and resilience. The framework is grounded in several established literatures, including emotion regulation theory, resilience research, music psychology, and music-evoked autobiographical memory. A brief illustrative example from practice is included only to clarify the model, not as empirical support for it. The model is intended as a heuristic and mechanistic conceptual architecture: it distinguishes analytically separable domains of musical experience while proposing plausible pathways through which they may become linked in resilience-related processes.

The article proceeds as follows. First, I clarify the conceptual need for an integrative account of music and resilience and situate the present framework against prior integrative models. Second, I outline the conceptual foundations on which the model rests. Third, I present the three-level model in detail, including an account of how the levels interact. Fourth, I discuss a brief non-evidentiary illustration and practical implications. Finally, I consider limitations and future research directions.

Three important qualifications frame the argument that follows. First, the present article is conceptual in nature: it develops a theoretical framework rather than reporting empirical findings, and the model’s claims await systematic empirical testing. Second, the structural dimensions described at Level 1 reflect features of tonal and tension-based musical traditions that are not universal across cultures; the framework should not be generalized globally without intercultural modification. Third, music is not inherently beneficial – its effects depend critically on context, regulatory strategy, and individual history, and under certain conditions musical engagement may intensify rather than alleviate distress. These qualifications are stated here directly so that the argument developed in the remainder of the article can proceed with appropriate confidence.

The broader aim is to show that music may be understood not merely as a vehicle for emotional expression, but as a structured experiential space through which individuals and groups may engage capacities central to personal and social resilience.

## Resilience, music, and the need for an integrative framework

While music has long been recognized as emotionally potent (Juslin and Västfjäll, 2008), research on its specific role in resilience and mental health remains fragmented and insufficiently integrated (McFerran et al., 2016). Some work emphasizes music’s role in emotion regulation; other work highlights autobiographical memory, identity, or community participation. These are all important contributions, yet they are not always integrated within a single explanatory framework. A more integrative account is needed – one that can explain how structural, intrapsychic, and interpersonal dimensions of musical experience interact.

The framework proposed here begins from a simple but important distinction: music is not itself resilient; rather, it affords experiences and practices that support resilience. More specifically, music may support resilience by shaping processes such as emotional regulation, meaning-making, autobiographical continuity, and interpersonal connection.

This position is compatible with contemporary resilience theory, which increasingly describes resilience not as invulnerability but as the activation of adaptive processes under challenge (Masten, 2001; Masten et al., 2021). From this perspective, music may be approached as one accessible, culturally ubiquitous, and often low-cost resource through which such adaptive processes can be supported.

### Prior integrative frameworks and the present contribution

Several influential frameworks have clarified important dimensions of the relationship between music and psychological functioning, but they tend to emphasize particular aspects of this relationship rather than integrating them into a single multi-level account of how music may support resilience.

Saarikallio (2011) examined music as a tool for emotional self-regulation throughout adulthood. Within this broader line of work, the Music in Mood Regulation framework identified seven key strategies through which individuals may use music to regulate their emotional states – entertainment, revival, strong sensation, diversion, discharge, mental work, and solace (Saarikallio, 2008). This empirically grounded framework is clinically useful, yet it primarily addresses regulatory strategies and functional goals rather than offering an explicit account of the structural properties of music, the role of autobiographical memory, or the social and dialogical dynamics emphasized at Level 3 of the present model. The present model addresses this gap by specifying structural properties of music that may contribute to regulatory effects at Level 1 and by connecting these to autobiographical meaning at Level 2. It further proposes that dialogical-social sharing at Level 3 may help interrupt maladaptive regulatory loops, such as ruminative cycling or unchecked discharge, that unmediated music use can otherwise sustain.

DeNora (2000) made a foundational contribution by showing how music can function in everyday life as a resource for mood regulation, self-organization, and social agency. Her account compellingly demonstrates that music is not merely passively consumed but actively used by individuals in the ongoing shaping of experience. At the same time, her framework is oriented primarily toward music’s practical and socio-cultural uses in everyday life, rather than toward a differentiated conceptual account of how musical structure, autobiographical meaning, and dialogical-social sharing may operate as distinct yet interacting levels in resilience-related processes. The present model extends this account by specifying why musical structure itself – not only its social and practical uses – may organize resilience-relevant experience in its own right. It further proposes that autobiographical meaning and dialogical recognition operate as analytically distinct yet interacting levels within a unified resilience framework, rather than as concurrent features of socio-musical practice.

Similarly, Ruud (1997) argued that musical experience plays a central role in the construction of identity and the maintenance of a coherent sense of self, drawing on narrative theory to show how musical memories anchor the person in time, place, and significant relationships. This perspective is highly relevant to the present argument, especially in relation to the autobiographical and identity-related dimensions of music. However, Ruud’s (1997) account does not explicitly develop a multi-level model that distinguishes between the structural experience of music, its autobiographical and symbolic significance, and its function as a medium of dialogical-social sharing in the context of resilience. The present model complements this account by explaining how temporal musical structure grounds the autobiographical and identity-related meanings Ruud foregrounds, and by specifying the dialogical-social conditions under which such meanings may be consolidated or deepened through interpersonal recognition – a process Ruud acknowledges but does not develop as a distinct analytical level.

Koelsch (2014) provided an important theoretical and neuroscientific account of music-evoked emotions, highlighting mechanisms such as expectancy, tension, emotional contagion, and reward, as well as the social functions of music. This work is especially relevant to the present model’s first level, as it helps explain how structural and perceptual features of music may shape affective experience in their own right. At the same time, Koelsch’s (2014) framework is oriented primarily toward the neural and psychological processes through which music induces emotion, rather than toward an explicitly integrative model of how musical structure, autobiographical meaning, and dialogical-social sharing may interact in resilience-related processes. In this sense, the present article builds on such accounts while extending the focus from emotion induction to the broader question of how music may become a resource for personal and social resilience. The present model complements this neural-affective account by asking how music-induced emotional processes may become linked to autobiographical meaning, and how that meaning may subsequently enter dialogical-social encounters in ways that cultivate both personal and social resilience – questions that fall beyond the primary scope of an account organized around emotion induction rather than resilience as a multi-level process.

Table 1 summarizes the key analytical distinctions between these prior frameworks and the present model. It is intended not to diminish these contributions, which are foundational, but to specify precisely where the explanatory gaps the present article is designed to address are located.

**TABLE 1:** Comparison of prior frameworks and the present three-level model.

Framework	Primary level of analysis	Structural (L1)	Autobio-graphical/ meaning (L2)	Dialogical-social (L3)	Explicit cross-level mechanism	Failure conditions specified
Saarikallio, 2008, 2011	Regulatory strategies	Implicit	Partial	Absent	No	Partial
DeNora, 2000	Socio-cultural practice	Implicit	Partial	Partial	No	No
Ruud, 1997, 2010	Identity/quality of life	Absent	Central	Partial	No	No
Koelsch, 2014	Neural/emotional mechanisms	Central	Absent	Partial	No	No
Present model	Multi-level resilience	Central	Central	Central	Yes	Yes

The present article builds on these foundational contributions. As Table 1 makes explicit, the distinctive claims of the present model are threefold: it treats structural, autobiographical, and dialogical-social dimensions as co-equal and interacting, rather than as primary and residual; it specifies the directional pathways through which movement across levels may occur; and it identifies boundary conditions under which these mechanisms may not support, or may actively undermine, resilience-related processes. Prior frameworks generally do not combine these three dimensions with an explicit account of cross-level transition and boundary conditions. The argument that follows is therefore not a refutation of prior work, but an integration and extension designed to answer questions the existing literature does not yet fully address.

Its central claim is that music may support resilience not only through its role in emotion regulation and identity-related processes, but also through the interplay between its temporal structure, its capacity to anchor autobiographical meaning, and its role as a medium of interpersonal recognition and shared reflection. What the present model adds, more explicitly than prior frameworks, is an account of how these domains may become linked within resilience-related processes: from structured musical experience, to autobiographical meaning, to dialogical-social recognition and support.

## Conceptual foundations

Several bodies of scholarship ground this model.

First, work on emotion regulation – especially Gross’s (1998, 2015) process-oriented account – suggests that regulation is not simply the suppression or elimination of feeling, but involves the modulation of emotional processes over time, including attention, appraisal, intensity, duration, and expression. Music is especially relevant here because it unfolds in time and can shape attention, expectation, arousal, and the temporal unfolding of emotion (Huron, 2006; Meyer, 1956). Musical form may thus be understood as providing a temporal analog for processes of emotional modulation.

Second, resilience research – especially the work of Masten (2001) and Masten et al. (2021) – emphasizes that resilience often emerges from ordinary adaptive systems rather than from rare heroic traits. This view invites us to consider music as an “ordinary” resource: widely available, culturally embedded, emotionally immediate, and woven into everyday life. Equally important for the present framework is Bonanno’s (2004) demonstration that resilience represents a distinct trajectory among responses to loss and adversity – not an absence of distress, but a pattern of relatively stable healthy functioning over time, despite transient disruptions following challenge. This trajectory-based understanding reinforces the model’s emphasis on temporal processes rather than static outcomes: music may be most relevant to resilience not as a one-time intervention but as a resource that supports functional continuity across time. Fredrickson’s (2001) broaden-and-build theory of positive emotions is also pertinent here. Fredrickson (2001) argues that positive emotional states broaden an individual’s momentary thought–action repertoire in ways that, over time, build durable personal and social resources. Applied to music, this suggests that music’s documented capacity to evoke positive affect – even in the context of difficult content, such as the pleasures of sadness in music (Sachs et al., 2015) – may make it a plausible vehicle for the kind of broadening that can support both personal and social resilience. Ungar’s (2008, 2012) social-ecological model of resilience is highly relevant here, as it frames resilience not as an individual trait alone, but as a process involving the capacity of individuals to navigate toward resources and the capacity of families, communities, and cultures to provide them in meaningful ways. This formulation offers an important theoretical foundation for the interpersonal and community dimensions developed at Level 3 of the present framework.

Third, music psychology has shown that music can evoke emotion through multiple mechanisms, including expectancy, memory, and social contagion. Work by Juslin and Västfjäll (2008) is especially helpful in showing that emotional responses to music are not reducible to a single pathway. Of particular importance for the present framework are expectation-based processes – which involve tension and release as described by Meyer (1956) and later by Huron (2006) – and memory-based processes, in which music becomes associated with personally significant episodes and meanings.

Fourth, research on music-evoked autobiographical memory (MEAM), particularly the work of Janata (2009), Janata et al. (2007), and Kaiser and Berntsen (2023), demonstrates that familiar music can function as a potent cue for personal memories, places, relationships, and felt aspects of identity. This is central to the present argument, because resilience depends not only on affective regulation but also on continuity of self, meaning-making, and the capacity to resituate painful experience within a broader personal narrative.

Fifth, research on music and emotion regulation suggests that musical engagement is not inherently beneficial. Its effects depend on the regulatory processes involved: when music is associated with adaptive strategies such as cognitive reappraisal, it may support wellbeing, whereas when it is associated with maladaptive processes such as rumination, expressive suppression, or certain forms of mood-congruent immersion, it may be associated with poorer outcomes (Chin and Rickard, 2014; Carlson et al., 2015). This distinction motivates the second and third levels of the model developed below, which specify the reflective and relational conditions under which musical engagement may support resilience.

Finally, Zaki and Williams’s (2013) interpersonal emotion-regulation framework distinguishes processes that depend on another person’s response from those that do not. Their account proposes that supportive responses can change perceived resources and appraisal. It provides a theoretical bridge for the Level 3 feedback pathway without establishing its music-specific effects.

Taken together, these literatures suggest that music’s relevance to resilience cannot be captured by a single mechanism. Rather, the evidence points toward the need for a layered framework that links temporal structure, autobiographical meaning, and interpersonal sharing within one conceptual account.

Beyond this layered complexity, what further distinguishes music from other potential resilience resources is the combination of properties it brings together within a single medium. Music is distinctive in its capacity to organize emotional meaning in largely non-propositional form: it can hold ambiguity, complexity, and unresolved feeling without requiring immediate translation into explicit verbal claims (Langer, 1942). Unlike other sound-based experiences such as ambient noise, music unfolds in time according to an internal structural logic – organizing expectation, tension, and release – that actively shapes attention, arousal, and emotional experience in ways that feel both immediate and structured (Huron, 2006). Music is often closely and spontaneously linked with autobiographical memory, and this connection can operate without deliberate effort: a familiar phrase can cue vivid recollections of places, relationships, periods of life, and emotionally significant experiences (Janata, 2009; Janata et al., 2007). And unlike most other personal resources, music is inherently social in its origins and uses – it is performed, shared, synchronized, and embedded in communal ritual across all known human cultures (Cross, 2009; Tarr et al., 2014). Music is also among the most universally practiced and accessible of the arts; it is documented in every known human culture (Mehr et al., 2019) and integrated into the daily lives of individuals across diverse social and economic contexts (Juslin and Laukka, 2004). It is this convergence – temporal structure, non-propositional emotional encoding, autobiographical anchoring, social embeddedness, and universal accessibility – that gives music its distinctive potential as a resilience resource, and that the three-level model developed below is designed to capture.

## A three-level model of music-based resilience

The model is summarized in Figure 1.

**FIGURE 1:**
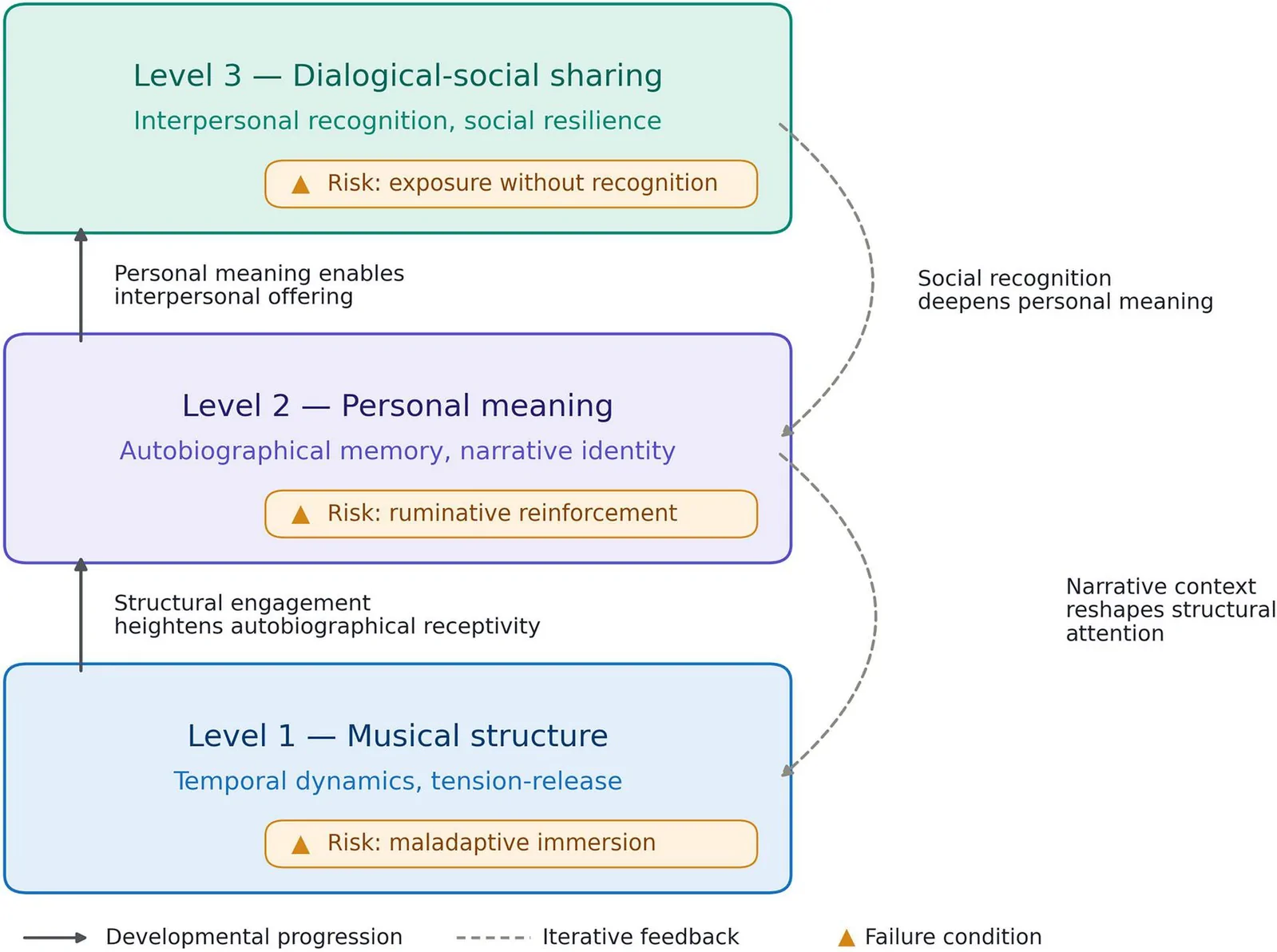
A three-level conceptual model of music-based resilience. Musical structure (Level 1), personal meaning (Level 2), and dialogical-social sharing (Level 3) are analytically distinct, interacting dimensions. Solid upward arrows depict possible forward pathways corresponding to P2 and P3; the upper dashed arrow depicts the feedback pathway specified in P4. P1 concerns immediate regulation within Level 1, rather than an interlevel arrow. The lower dashed arrow represents an additional exploratory pathway through which narrative context may reshape structural attention. “Developmental progression” denotes a possible sequence of engagement, not required stages. Arrow labels express theoretical predictions rather than established causal effects; risk markers identify potential adverse processes, not inevitable outcomes. Table 2 specifies the proposed mediators and moderators. All resilience-related pathways require empirical evaluation.

### Level 1: musical structure

At the first level, the focus is on music itself as structured temporal experience. Musical structure – especially in tonal traditions, though not exclusively so – organizes time through processes such as tension and release, instability and resolution, movement and pause, expectation and delay, consonance and dissonance. These are not merely technical features; they are also lived dynamics, experienced by listeners with and without formal musical training (Huron, 2006; Meyer, 1956).

From a resilience perspective, the significance of these dynamics lies in the fact that music can contain tension, instability, and unresolved movement without losing form. A dissonance does not end the piece. Suspense does not necessarily imply breakdown. Instead, tension can be held, developed, and transformed within an unfolding temporal whole. Music may therefore provide a vivid experiential analog of resilience-related processes: it does not eliminate tension, but can give tension temporal shape.

This is especially relevant to emotion regulation, which is sometimes understood primarily as calming down or reducing emotional intensity. Yet resilience-relevant regulation may depend less on feeling less than on responding flexibly to situational demands – at times staying with difficult emotion and modulating it, at others reducing its intensity or redirecting attention (Aldao et al., 2010; Bonanno and Burton, 2013). Rumination – repetitive, passive focus on distress and its causes – showed the largest association with psychopathology of the six strategies examined by Aldao et al. (2010). Several theoretical frameworks help specify the mechanisms through which this temporal-structural engagement may produce genuine regulatory effects – not only as a phenomenological parallel to coping, but as an engagement of the very processes coping requires. From the perspective of embodied and enactive cognition, musical experience is not reducible to passive perception; rather, the listener’s body and attentional system actively track and anticipate musical events in time (Clarke, 2005). Stern’s (2010) concept of vitality dynamics is especially relevant here: dynamic patterns of movement, time, force, space, and directionality – qualities that are central to musical structure and experience – constitute a fundamental layer of felt experience through which arousal, motivation, and intersubjective attunement are organized. On this account, musical engagement is not merely analogous to emotional regulation; it may recruit temporal, arousal-shaping, and attentional processes that are also central to the emotion regulation process itself. A related account can be derived from attentional theories of emotion regulation: by directing and sustaining attention through structured temporal events, music may provide a low-stakes context for practicing the regulatory skills of staying present with rising affect, inhibiting premature avoidance, and waiting for resolution. These processes – directed attention, inhibition of escape, and toleration of unresolved arousal – are among the processes that adaptive coping may require when situational demands call for engagement rather than disengagement (Gross, 1998, 2015; Bonanno and Burton, 2013). Whether engagement with Level 1 in fact produces durable changes in these capacities remains an empirical question. The present claim is the more modest one: that the structural properties of music engage the right systems, in plausible ways, to make such transfer possible – and that this possibility is sufficiently grounded to warrant systematic empirical investigation.

Existing empirical findings are consistent with these theoretical claims, even in the absence of direct tests of the present model. Neuroimaging research has shown that intensely pleasurable musical experiences engage the striatal dopaminergic system, with anatomically distinct responses during anticipation as the music unfolds and during the experience of peak emotional arousal (Salimpoor et al., 2011). Complementary work on the psychological mechanisms of musical expectancy indicates that systematic variations in structural predictability can produce measurable changes in continuous perceived emotional arousal (Sauvé et al., 2018), consistent with the arousal-regulatory engagement the present model proposes. These findings do not verify the model’s resilience-related claims, but they show that Level 1 engagement recruits anticipatory and arousal-related processes of the kind the framework identifies as resilience-relevant; whether such engagement develops regulatory capacity remains untested.

Music may offer a contained simulation of this process by allowing emotional movement to be experienced within a bounded and aesthetically organized form. When engaged intentionally, music may therefore become a space for practicing guided attention: noticing tension as it arises, staying with it without premature escape, and experiencing release when it comes. Such engagement does not in itself amount to resilience, but it may support capacities that are relevant to resilient functioning.

At this level, three resilience-related capacities are especially salient:

#### Emotion regulation

Music offers a contained experience of affective movement. Through recurring patterns of tension and release, listeners may encounter a manageable analog of emotional arousal and recovery. This helps explain why music can be used not only to change mood, but also to remain with difficult affect in ways that feel structured rather than overwhelming.

#### Tolerance for complexity and ambivalence

Human experience is rarely affectively simple. People often feel sadness and hope, fear and longing, grief and gratitude simultaneously. Music may be especially suited to holding such complexity because it does not need to reduce experience to a single verbal proposition. It can sustain ambiguity, layered feeling, and unresolved motion without forcing premature closure.

#### Continuity under uncertainty

Musical continuity is especially important here. Even when harmonic resolution is delayed or musical tension intensifies, music typically continues to unfold. This continuity can function as an experiential image of persistence under uncertainty: movement continues even when certainty is not yet available. The claims developed in this section concern perceived temporal dynamics in listener experience – especially in relation to tonal and tension-based musical forms – rather than a universal property of all musical systems.

### Level 2: personal meaning

The second level begins when musical structure meets biography. Music becomes more than an organized sequence of sounds when it is linked to a person’s life history, relationships, losses, hopes, or self-understanding. At that point, it may function as a narrative anchor. This is especially true of songs, whose lyrics may further deepen and articulate personal meaning.

This level is indispensable to the framework because resilience is not only a matter of enduring affect; it also involves the construction of meaning. A difficult experience often becomes more bearable when it can be understood as part of a broader story, relationship, identity, or system of values (McAdams, 2001). Music is especially powerful in this regard because it can carry both non-verbal feeling and autobiographically saturated memory (Janata, 2009; Janata et al., 2007; Kaiser and Berntsen, 2023). Songs may be particularly potent because lyrics add an explicit verbal-symbolic layer to musical experience. Whether this lyrical dimension facilitates autobiographical narrative organization remains an empirical question.

Familiar music often functions as a condensed capsule of meaning. A musical phrase, motif, or song may reconnect a listener with a place, a loved one, an earlier sense of self, a period of hardship, or a source of strength. In such moments, the music is not merely heard; it is re-experienced as part of an emotionally and narratively charged world (Ruud, 2010; DeNora, 2000).

This helps explain why music, and especially songs, can become resources in moments of crisis. A meaningful piece of music can be internally retrieved and used as a portable anchor. It may restore continuity, evoke hope, recall relationships, or help a person reorganize feeling around a symbolically charged memory. Songs may be especially effective in this respect because lyrics can provide a verbal dimension through which emotional experience may become more symbolically accessible and narratively organized, sometimes allowing individuals to return to a phrase or line as a concise symbolic resource. While Ali and Peynircioğlu (2006) show that lyrics and melodies both contribute to emotional communication in songs – with melodies generally dominant in their studies – the specific role of lyrics as semantic anchors for autobiographical meaning remains a theoretical proposal requiring empirical investigation.

At the same time, autobiographically meaningful music may also intensify grief, longing, or ruminative return when memories are repeatedly activated without reflective or relational processing. The model helps address this risk by distinguishing private autobiographical activation from structured attentional engagement at Level 1 and dialogical-social recognition at Level 3.

This level also helps explain why the emotional meaning of music cannot be inferred from structure alone. Broad musical conventions matter: listeners often share expectations related to tempo, mode, melodic contour, and tension. Yet autobiographical memory can significantly shape musical experience. A piece of music that might conventionally be experienced as sad may acquire comfort, gratitude, or even joy through its association with a positive autobiographical memory (Janata et al., 2007). Therefore, when someone chooses meaningful music, the crucial question is not only “What does the music express?” but also “What does this music mean to this particular person?”

### Level 3: dialogical-social sharing

The third level concerns contexts in which personally meaningful music is shared with others and becomes a medium of interpersonal recognition, mediated disclosure, and possible mutual support. The term “dialogical” is used here in a broad sense inspired by dialogical perspectives in which personhood is shaped through relation, response, and address (Bakhtin, 1981; Buber, 1958). The claim is not that every instance of shared music becomes dialogue in a strong dialogical sense, but that the sharing of music can create conditions for a distinctive form of interpersonal encounter.

This level is important because resilience is not only an intrapsychic process. It is also shaped by relationships, communities, and the ability to experience oneself as seen, heard, and connected to others (Ungar, 2008, 2012). Research on music and social bonding is also relevant here, suggesting that musical engagement can support interpersonal attunement, shared attention, and a sense of connection (Tarr et al., 2014). Ansdell’s (2014) account of how music helps in music therapy and everyday life is also instructive here: it emphasizes that music becomes helpful through the relational and situational conditions in which it is used, rather than through musical properties alone – a position consonant with the boundary conditions specified below.

In educational, therapeutic, and community settings, the sharing of meaningful music can open a distinctive interpersonal space. People may disclose memories, vulnerabilities, values, losses, or hopes through music in ways that feel less exposed than direct self-description. A song can function as a mediating object: it gives expression to something deeply personal, but indirectly – through sound, melody, and association rather than explicit verbal declaration alone.

What emerges in such moments is more than personal expression. Sharing music and the personal stories attached to it can also become a meaningful act of offering. Participants do not merely reveal something about themselves; they give others access to an aspect of experience that might otherwise remain difficult to communicate. In this sense, music-sharing may become a prosocial act through which one person’s resource becomes available, at least in part, to others. For the person offering music, this act may also carry a sense of agency – of having something of value to contribute – which is itself relevant to resilience.

At the group level, such processes may foster belonging and mutual recognition. One person’s music may resonate with another person’s experience, even across differences in biography, worldview, or background. The result is not necessarily agreement or sameness, but a widened space of recognition in which plurality can be held without immediate erasure of difference.

A structurally important feature of this level is its fundamentally reciprocal character. Music-based sharing involves not only giving – offering one’s resource, story, and symbolic world to others – but also receiving: being genuinely heard, recognized, and responded to by another person. These two movements are not merely symmetrical; they activate distinct resilience-relevant processes. For the person who gives, the act of offering meaningful music to others may reinforce a sense of agency, self-efficacy, and prosocial purpose – a felt sense of having something of value to contribute, which is itself protective under conditions of adversity. For the person who receives – who allows another’s music and story to matter, and who responds with recognition – the encounter may deepen attunement, extend empathic capacity, and sustain the relational conditions on which social resilience depends. For the sharer who is received, the experience of being genuinely heard can consolidate personal meaning in ways that internal processing alone does not reliably achieve: recognition by another may strengthen autobiographical coherence and restore a sense of being connected to a larger human world. This bidirectionality aligns with Buber’s (1958) dialogical principle that genuine encounter requires openness on both sides, and with Ungar’s (2008, 2012) social-ecological model, which frames resilience as depending not only on the individual’s capacity to seek support, but on the community’s capacity to provide it in personally meaningful ways. Music-sharing, understood in this light, is not a one-directional act of disclosure but a reciprocal process through which both personal and social resilience may be simultaneously cultivated.

This level may be especially relevant in contexts marked by social fragmentation, stress, or division. Music does not automatically produce empathy or cohesion, nor should its social potential be romanticized. Nevertheless, work on musical meaning and mechanisms of social bonding suggests that musical interaction can facilitate shared intentionality, affiliation, and social closeness (Cross, 2009; Tarr et al., 2014). Whether sharing personally meaningful music is experienced as safer or less defensive than comparable verbal discussion remains an untested prediction of the present model. Under supportive relational conditions, the model further proposes that such sharing may help transform private coping into shared resilience.

### Cross-level dynamics

The three levels described above are analytically distinct but functionally interrelated. They should not be understood as strictly sequential stages that a listener necessarily passes through in a fixed order. Rather, they represent different dimensions of musical experience that may be activated simultaneously, in different combinations, or with different degrees of salience depending on context, intention, and individual history.

That said, a developmental logic is often discernible. In practice, engagement with Level 1 — intentional attention to the structural dynamics of music — may serve as a gateway that heightens receptivity to personal and interpersonal meanings. A listener who becomes aware of tension, suspension, or release may then connect those dynamics to an autobiographical memory or life struggle (Level 2). In turn, a person who has identified a meaningful piece of music may later share it with others (Level 3). Proposition 1 specifies a proposed regulatory pathway within Level 1; Propositions 2 and 3 specify the forward transitions between levels. The movement across levels is therefore best understood as a possible developmental progression rather than a necessary sequence.

The levels also interact in the other direction: responsive sharing at Level 3 may deepen personal meaning at Level 2 (Proposition 4). Narrative context may, in turn, reshape attention to musical structure at Level 1. Figure 1 includes this latter possibility as an additional exploratory feedback pathway; it is not separately formalized in P1–P4. The architecture is therefore iterative rather than strictly linear.

Critically, the model also specifies conditions under which music may fail to support resilience or may intensify distress. At Level 1, persistent ruminative engagement may undermine containment. Discharge-style listening has been associated with higher anxiety and neuroticism, particularly among males (Carlson et al., 2015); these associations do not establish that discharge or mood-congruent listening is invariably harmful. More broadly, the wellbeing benefits of music engagement appear to depend on whether adaptive strategies such as cognitive reappraisal, rather than expressive suppression, operate as the primary regulatory mechanism (Chin and Rickard, 2014), and research identifies further risks involving avoidance and rumination (Garrido and Schubert, 2013; Saarikallio et al., 2015). The model therefore predicts that responsive sharing may be particularly useful when private music-based coping has become ruminative, by introducing interpersonal resources that support a changed appraisal (Zaki and Williams, 2013). This is a conditional prediction, not an established effect. At Level 3, disclosure without an attuned or validating response may produce exposure without recognition. Repeated discussion that reinforces distress without opening alternative perspectives may likewise fail to help. The regulatory strategy, relational context, and manner of reception are therefore proposed influences on outcomes, rather than guarantees of success or failure.

### Core propositions

To make the model’s predictive content explicit, the proposed relationships can be stated as four core propositions. Each distinguishes a predictor, a proposed mediator, an outcome, and moderating conditions. These propositions synthesize existing theory and evidence; they have not been directly tested as an integrated model. P1 concerns immediate regulation during listening, P2 and P3 concern forward transitions, and P4 concerns feedback from sharing to personal meaning. Table 2 summarizes the propositions. Figure 1 shows the interlevel architecture and potential risks; its caption explains the correspondence with P1–P4.

**TABLE 2:** Core propositions of the three-level model.

Proposition	Predictor → outcome	Proposed mediator	Moderators and potential failure conditions	Theoretical grounding
P1	Guided structural attention → manageable affective engagement during listening	Sustained attentional tracking	Strategy–context fit; starting arousal. Risk: persistent rumination or overwhelming arousal.	Gross, 1998, 2015; Huron, 2006; Clarke, 2005; Stern, 2010; Bonanno and Burton, 2013
P2	Guided structural attention to familiar music → narrative integration	Accessibility and elaboration of autobiographical memories	Familiarity; personal significance; reflective opportunities. Risk: ruminative repetition without integration.	Janata, 2009; Janata et al., 2007; McAdams, 2001; Garrido and Schubert, 2013
P3	Sharing meaningful music and its story → recognition, belonging and agency	Reduced perceived disclosure threat; willingness to disclose	Relational safety; facilitation; listener openness. Risk: dismissive, intrusive or evaluative reception.	Buber, 1958; Ungar, 2008, 2012; Rentfrow and Gosling, 2006; Boer et al., 2011; Liu and Wang, 2026
P4	Responsive listener behaviors → integration and reduced subsequent rumination	Sharer’s felt understanding and perceived support, enabling reappraisal	Prior rumination; relational trust. Risk: invalidation or reinforcement of repetitive distress.	Zaki and Williams, 2013; Bonanno, 2004; Ungar, 2012

#### Proposition 1 (Level 1 → immediate regulation)

Intentional attention to musical tension, suspension, and release is predicted to support manageable affective engagement during listening through sustained attentional tracking of musical events (Huron, 2006; Clarke, 2005; Stern, 2010). Gross’s (1998, 2015) attentional-deployment account grounds this pathway: directing attention is itself a means of shaping emotional experience. Proposed moderators are the regulatory strategy’s fit with current goals and demands and the listener’s starting arousal. The pathway may be weakened or reversed by persistent ruminative engagement or overwhelming arousal; mood-congruent listening alone does not imply failure (Carlson et al., 2015; Bonanno and Burton, 2013). Transfer to durable regulatory capacity remains a separate longitudinal question.

#### Proposition 2 (Level 1 → Level 2)

Guided structural attention to familiar music is predicted to increase the accessibility and elaboration of autobiographical material, particularly when the music carries prior personal significance, thereby facilitating narrative integration. Music-evoked memory research establishes that music can cue autobiographical material, including spontaneously (Janata, 2009; Janata et al., 2007); narrative theory links such material to the organization of identity (McAdams, 2001). The present model extends these accounts by proposing that structured attention can make relevant cues available for reflection. This attentional contribution is untested and is distinct from the prediction that personally significant music supports greater integration than autobiographically neutral music. Proposed moderators are familiarity, personal significance, and opportunities for reflection. Memory activation need not produce integration: persistent ruminative repetition may weaken or reverse the pathway (Garrido and Schubert, 2013; Saarikallio et al., 2015).

#### Proposition 3 (Level 2 → Level 3)

Sharing personally meaningful music and its associated story should, under adequate relational conditions, support felt recognition, belonging, and prosocial agency through reduced perceived threat of disclosure and greater willingness to communicate significant experience. Music’s proposed role as a mediating object explains why disclosure may feel less direct. Buber’s (1958) emphasis on reciprocal openness and Ungar’s (2008, 2012) account of culturally meaningful social resources motivate the relational conditions under which disclosure may become supportive encounter. Proposed moderators are relational safety, facilitative structure, and the listener’s openness. These theories motivate, but do not establish, an advantage over comparable verbal-only sharing. Dismissive, intrusive, or evaluative reception may instead produce exposure without recognition.

Existing evidence supports components of this pathway without establishing it as a whole. Musical preferences convey identity- and value-relevant information in interpersonal perception (Rentfrow and Gosling, 2006), perceived similarity in musical taste fosters social attraction through inferred value similarity (Boer et al., 2011), and music can shape the content and form of self-disclosure (Jensen, 2001). Relationally framed, responsive music sharing has increased perceived social presence relative to informational posting, although music, disclosure, and responsiveness were manipulated together (Liu and Wang, 2026), and qualitative evidence from a music-listening group with older men shows that shared self-selected music did not automatically elicit self-disclosure, underscoring the role of interpersonal and contextual conditions (Lindblad, 2021). None of this directly tests reduced disclosure threat as the mediating process. Of the four propositions, P3 therefore remains the least directly supported and the most important target for experimental testing.

#### Proposition 4 (Level 3 → Level 2, feedback)

Responsive listening behaviors during musical sharing are predicted to support autobiographical integration and reduce subsequent rumination through the sharer’s felt understanding and perceived access to support, which may enable reappraisal. Zaki and Williams’s (2013) account of response-dependent interpersonal regulation supplies the theoretical basis for this mechanism. Here, the other person’s observable response is the predictor, the sharer’s experience is the mediator, and subsequent integration and rumination are outcomes. Proposed moderators are prior rumination and relational trust. Benefits may be greater when private coping has remained ruminative, but responses that invalidate experience or reinforce repetitive distress may weaken or reverse the pathway. Bonanno’s (2004) trajectory perspective and Ungar’s (2012) social ecology motivate examining whether these proximal changes contribute to sustained adaptation; they do not establish the music-specific feedback effect.

These propositions specify conditional predictions, not reliable effects or necessary stages. The comparisons in Directions for Future Research identify how their distinct mechanisms and outcomes could be tested.

## Illustrative, non-evidentiary example

The following composite illustration is drawn from the type of structured group process the present model is designed to describe. It represents no specific individual and is presented purely as a heuristic device to make the model’s architecture more legible. It should not be read as empirical evidence for the framework’s claims.

Consider a participant in a group workshop focused on professional resilience – for example, a training program for educators navigating institutional change, personal strain, and role uncertainty. In a preliminary guided listening exercise, participants are invited simply to listen to a short instrumental piece and to notice, without judgment, any shifts in tension, movement, suspension, or release that they perceive in the music itself. One participant remarks afterward that she noticed a section of unresolved tension that grew in intensity, followed by a quiet return to the opening material, which she experienced as a release. Remaining attentive to this unresolved passage – rather than mentally moving away from it, and without escaping into detachment or becoming overwhelmed – felt unexpectedly meaningful. This illustrates Level 1 engagement: intentional, guided attention to musical structure as structured temporal experience.

In a subsequent exercise, participants are invited to bring a song that has mattered to them during a difficult period. The same participant brings a song she associates with a time several years earlier when she had considered leaving her profession. The song, she explains, had kept returning to her during that period – not because its lyrics directly described her situation, but because it had been playing on the day she decided to stay. The music had become a condensed capsule of that moment of choice. This illustrates Level 2: the song functions as an autobiographical anchor, reconnecting the participant with a sense of agency and self-continuity at a moment when her current difficulties might otherwise feel disconnected or without precedent in her own story.

When she shares this in the group – playing a brief excerpt and narrating its meaning – the interpersonal field changes. A colleague responds not by offering advice, but by recognizing something in her account. He says that he had not expected to understand her situation more fully than he did through her verbal explanation alone, but that the music allowed him to feel something of its emotional weight. Within the terms of the present model, this moment illustrates Level 3: dialogical-social recognition. For the participant, being received in this way – heard not only propositionally but also affectively – may help consolidate the personal meaning she had previously held privately. Recognition by another can thus feed back into Level 2, deepening autobiographical coherence.

This composite sequence illustrates the model’s cross-level architecture in an applied but non-evidentiary form. It also clarifies the model’s boundary conditions. Had the group environment lacked sufficient relational safety for genuine reception – had the colleague’s response been evaluative, dismissive, intrusive, or merely polite – the act of sharing might have produced exposure without recognition. Such an outcome would represent not a failure of music per se, but a failure of the dialogical conditions required for Level 3 to support resilience.

## Discussion and practical implications

In education, music may be used not only for artistic appreciation but also as a structured means for reflective learning, emotional literacy, and relational dialogue. Inviting students to notice musical tension and release, to connect music with autobiographical memory, and to share music-based narratives may enrich both psychological insight and group cohesion. The three-level architecture may offer educators one way of sequencing such activities: beginning with guided structural listening, moving to personal reflection and narrative, and progressing to interpersonal sharing.

In therapy and psychosocial intervention, the model suggests that music-based work may benefit from distinguishing among structural listening, personal meaning-making, and dialogical sharing. These dimensions may help to formulate goals more clearly: for example, whether the aim is to support regulation, strengthen autobiographical continuity, or facilitate interpersonal recognition. The model is intended as a conceptual aid rather than a prescriptive protocol.

In community and intergroup contexts, music may serve as a medium through which people communicate aspects of identity, vulnerability, and care across social, cultural, or ideological differences. Because the sharing of meaningful songs does not depend entirely on direct propositional argument, it may create forms of encounter that are less ideologically rigid than direct verbal exchange. This may be especially relevant in contexts of social division or post-conflict recovery, where the conditions for open dialogue are fragile.

In resilience-oriented programming, the model suggests one possible design for interventions that move intentionally across levels: from guided listening, to narrative reflection, to interpersonal exchange. Such progression may help transform brief emotional experiences into lasting personal and social resources.

The practical implications discussed here follow from the conceptual architecture of the model rather than from empirical evaluation. Systematic evaluation of such applications remains a task for future research.

## Limitations and critical considerations

Several additional cautions are necessary, beyond the three qualifications stated in the Introduction.

First, the cultural scope of the framework warrants further comment. Beyond the core caveat noted above, a broader intercultural perspective is needed, one that engages ethnomusicological and cross-cultural approaches to the ways different musical traditions organize temporal experience, emotional meaning, and social participation. Such work would be essential for testing the generalizability of the structural claims made at Level 1.

Second, the social use of music should not be idealized. Shared music can create solidarity, but it can also reinforce in-group boundaries and exclude those whose musical associations differ. For music to support genuine dialogue rather than mere affiliation, the conditions of listening and exchange matter greatly. The dialogical ideal described at Level 3 requires not only the presence of music, but also facilitative structures, trust, and a willingness to be changed by the other’s experience.

Third, the proposed moderators do not exhaust individual differences in musical responsiveness, which are likely to be substantial. Factors such as musical sophistication, absorption, alexithymia, trauma history, and cultural background will shape how individuals engage with music at each level. Future work should attend to these moderating variables. In particular, the reciprocal and dialogical mechanisms proposed at Level 3 currently rest more on theoretical synthesis and practice-informed observation than on a mature body of direct empirical testing.

## Directions for future research

The framework developed here opens several lines of inquiry, and its value as a theoretical contribution will ultimately depend on whether its components can be operationalized and tested empirically. The following directions are proposed with this goal in mind.

A first direction is the experimental decomposition of P1–P4. For P1, guided structural attention could be compared with an active listening control matched for music, duration, and researcher contact. The prediction is more manageable affective engagement during listening, mediated by sustained tracking rather than simply greater liking; later transfer requires separate assessment. For P2, attentional guidance and autobiographical significance should be varied separately, matching or accounting for familiarity and liking. Guided attention is predicted to increase integration through memory accessibility and elaboration, particularly for personally significant music when reflection is available. Memory vividness or intensity alone would not establish narrative integration. For P3, playing a meaningful song and sharing its story could be compared with verbal-only sharing of comparable content and duration. The prediction is lower perceived disclosure threat and greater willingness to disclose, contributing to felt recognition and belonging under supportive relational conditions. For P4, responsive musical sharing could be compared with private reflection or a suitable conversational control. Observable listener responses should precede assessment of the sharer’s felt understanding and support, followed by changes in rumination and integration. The predicted indirect effect may be stronger when prior rumination is higher, conditional on relational trust. Repeated measurements and appropriate controls would be needed to distinguish mediation from concurrent association.

A related question concerns lyrics as possible semantic anchors at Level 2. Comparing a song with an instrumental version and a spoken rendition of its lyrics, while accounting for familiarity and liking, could test whether the combined musical-verbal form supports greater articulation of personal meaning. This is an additional prediction, rather than evidence that lyrics or Level 2 are necessary for all music-related benefits.

A second direction concerns qualitative and process-based research. Qualitative studies may explore how participants describe transitions from musical experience to personal narrative to social connection, and whether the level architecture is recognizable in lived accounts. Such research would be especially valuable in capturing the subjective texture of cross-level transitions – particularly the movement from private meaning at Level 2 to interpersonal recognition at Level 3 – which experimental designs may not fully illuminate.

A third direction concerns the conditions under which music-sharing in group settings produces genuine dialogue rather than mere performance or self-protection. Under what conditions does sharing a meaningful song increase trust, empathy, or mutual support? When does it fail or produce unexpected negative effects? And how do group norms, facilitation practices, and relational safety shape whether music becomes a site of genuine dialogical encounter? Research on facilitation practices and group dynamics in music-based interventions is needed to address these questions systematically.

A fourth direction concerns cross-cultural generalizability. Comparative work across musical traditions and cultural contexts would be especially valuable in testing the scope of the structural claims made at Level 1, which are grounded primarily in tonal and tension-based musical forms. Ethnomusicological and cross-cultural perspectives will be essential here.

Finally, longitudinal and intervention-based research may examine whether the immediate processes proposed in P1–P4, repeated over time, contribute to lasting changes in resilience-related capacities – including emotion regulation, autobiographical coherence, sense of belonging, and self-efficacy – and whether such gains generalize beyond the musical context itself. Assessing maintained or recovered functioning under adversity would help distinguish resilience from short-term changes in mood, perceived support, or narrative coherence.

## Conclusion

Music does not make suffering disappear, nor does it guarantee resilience. It does, however, offer something important: a structured, embodied, and widely accessible medium through which people may engage capacities that matter for adaptation under strain.

At the level of structure, music may provide an experiential analog of tension, containment, movement, and release. At the level of personal meaning, it becomes linked to autobiography, identity, and narrative continuity. At the dialogical-social level, it may become a medium of recognition, offering, and mutual support.

What distinguishes the present framework from prior accounts is its explicit theorization of all three levels within a single model, together with an account of how the levels may interact. By specifying not only what each level contributes, but also how movement across levels may occur, the model offers a more integrated account of music as a resource for personal and social resilience. Crucially, the model also specifies conditions under which music may fail to support resilience, thereby identifying boundary conditions that sharpen, rather than weaken, its theoretical claims.

Seen in this way, music is valuable not only because it expresses feeling, but because it may help people remain with feeling without becoming overwhelmed, symbolize experience without reducing it to a single interpretation, and enter interpersonal sharing through forms other than propositional argument alone.
